# Predictors for Increasing Eligibility Level among Home Help Service Users in the Japanese Long-Term Care Insurance System

**DOI:** 10.1155/2013/374130

**Published:** 2013-09-09

**Authors:** Kuniyasu Kamiya, Kenji Sasou, Makoto Fujita, Sumio Yamada

**Affiliations:** ^1^Program in Physical and Occupational Therapy, Nagoya University Graduate School of Medicine, Nagoya 461-8673, Japan; ^2^Welfare Department, CO-OP AICHI Consumer Cooperative Society, Toyokawa 442-0838, Japan; ^3^Welfare Department, CO-OP KANAGAWA Consumer Cooperative Society, Makoto, Yokohama 225-0014, Japan; ^4^Department of Rehabilitation Science, Nagoya University Graduate School of Medicine, 1-1-20 Daiko-Minami, Nagoya 461-8673, Japan

## Abstract

*Objectives*. This cross-sectional study described the prevalence of possible risk factors for increasing eligibility level of long-term care insurance in home help service users who were certified as support level 1-2 or care level 1-2 in Japan. *Methods*. Data were collected from October 2011 to November 2011. Variables included eligibility level, grip strength, calf circumference (CC), functional limitations, body mass index, memory impairment, depression, social support, and nutrition status. *Results*. A total of 417 subjects (109 males and 308 females, mean age 83 years) were examined. There were 109 subjects with memory impairment. When divided by cut-off values, care level 2 was found to have higher prevalence of low grip strength, low CC, and depression. *Conclusions*. Some potentially modifiable factors such as muscle strength could be the risk factors for increasing eligibility level.

## 1. Introduction

The rising cost of long-term care (LTC) for elderly has become a growing concern. According to a forecast released by the Organization for Economic Cooperation and Development (OECD), more than twice the cost of LTC will be needed in 2050 [1]. Indeed, Japan has been experiencing rapid growing of aging population which the world has never experienced, and the cost of LTC insurance system doubled in only 10 years since the system started [2].

The Japanese LTC insurance system consists of 7 eligibility levels, including 2 support levels and 5 care levels. Among support levels, the purpose to utilize the service is basically prevention of increasing to care level. In contrast, care level 1 or higher need some help for activities of daily living, and care level 3 or higher need totally care for ambulation or clothing. In addition, the cost of care service for care level 3 or higher accounts for two-thirds of total LTC cost, although the number of people is less than 40%. For this reason, prevention of increasing to care level 3 or higher is important.

To prevent increasing eligibility level with efficient intervention, it is important to investigate the risk factors that enable to find out the high-risk subjects. However, previous longitudinal studies investigated unmodifiable factors such as gender, economic status, and caregivers as independent factors [3, 4]. Modifiable factors such as muscle strength or mass, nutritional status, and depression have been identified as risk factors for disability, hospitalization, or death in healthy community-dwelling elderly [5–14]. We hypothesized that these modifiable factors could be applied to risk factors for increasing eligibility level among those who were certified eligibility level.

The purpose of this study was therefore to reveal the prevalence of potential risk factors for increasing eligibility level from the cross-sectional observational study among those who were certified care level 2 or lower.

## 2. Methods

### 2.1. Design

The sample for this study was drawn from community-dwelling elderly who utilized home help services provided by Consumers' Cooperatives (Co-op) Aichi and Kanagawa in Japan. The recruit and survey were conducted in October and November, 2011. The study protocol was approved by the ethics review board of the Nagoya University Graduate School of Medicine (approval no. 1274). Written informed consent was obtained from each of the subjects prior to enrollment in the study. All data were organized and centralized at the data center of the Yamada laboratory at the Nagoya University Graduate School of Medicine.

### 2.2. Subjects

The subjects were drawn from individuals aged ≥65. Inclusion in the study required a certified LTC support level of 1-2 or care level of 1-2. Subjects who were difficult to communicate with were excluded.

The sample size was calculated from the track record of home help service users in Co-op Kanagawa from March 2007 to March 2010. The incidence rates of increasing to care level 3 or higher in 3 years were 4.3% in support level 1, 4.1% in support level 2, and 7.2% in care level 1. In contrast, care level 2 had 27.7% of remarkable high rate. Therefore, we assumed care level 1 or lower to be one group and planned to recruit the same numbers of subjects from care level 1 or lower and care level 2. Then, we assumed that the hazard ratio of increasing to care level 3 or higher of those who had a risk factor to those who did not was 2.0. We considered the lowest quartile of each parameter such as grip strength or calf circumference (CC) as a cut-off value for determining a risk factor. At least 359 subjects are required for each arm to provide 80% power to detect a hazard ratio of 0.5 using a 2-sided significance level of 0.05 by chi-square test. After considering maximum 20% for the dropouts or unsuitable for analysis, we have expected that approximately 449 subjects are needed to be enrolled in this study.

*Demographic Characteristics*. The data of eligibility level, service utilization, medical history, and family configuration were provided by care managers, who visit those who utilize LTC services once a month and manage their care plans.

*Eligibility Level*. Initial certification of eligibility levels in LTC insurance system is conducted when individuals or their families send in the application to the municipal government. Officials assess the applicant's physical and mental health, and the time required for nursing care is calculated. Then, the LTC certification committee rates the eligibility level based on this assessment. For example, if the required time for nursing care is less than 32 minutes, the applicant is to be support level 1. And if the time is more than 110 minutes, the applicant is to be care level 5. People in support levels 1-2 are able to independently perform basic activities of daily living, such as ambulation or clothing, and they are considered to be needing some support to prevent increasing eligibility level by physical or mental impairments. People in care levels need assists for performing basic activities of daily living. Typically, people in care level 1-2 are able to walk independently, while people in care level 3–5 have difficulty in walking alone. As a general rule, eligibility levels need to be updated within 12 months.

*Measures.* Measurements were performed in each subject's home. Body weight was measured using a digital weight scale (BC-301-SV; Tanita Co, Tokyo, Japan).

As an alternative indicator of height, demispan was measured in the supine position. Demispan was defined as the distance from the clavicular notch of the sternum to the opposite fingertip while the arm is laterally outstretched [15]. In measuring the height of the elderly, demispan resolves problems such as the errors caused by kyphosis or difficulty in standing. A previous study measured demispan in the seated position [15]. However, we measured demispan in the supine position and height in the standing position on a separate group of subjects aged ≥50 years without kyphosis (75 males and 68 females). The estimated formula was *y* = 1.38*x* + 45.3 (cm) (*r* = 0.77,  *P* < 0.01) for males and *y* = 1.28*x* + 49.6 (cm) (*r* = 0.70, *P* < 0.01) for females.

Calf circumference is an index of screening the risk for disability [9]. CC was measured at the point of greatest circumference while the subject was supine with the knee flexed at 90°. Measurements were made for both legs and the greater value (in mm) was used as the index for CC.

Handgrip strength was measured with the Jamar dynamometer set at the second handle position. The participant sat with the wrist in a neutral position and the elbow flexed at 90° [16]. The grip strength of each hand was measured twice, and the highest value (in 0.1 kg) was used as the index of handgrip strength.

Prior to administering the survey of home help service users, the principal investigator provided and handled training sessions for the above-described measurements to care managers or persons in charge of providing services in the Co-op Aichi and Co-op Kanagawa. One hundred and twenty-five examiners, including 56 from Co-op Aichi and 69 from Co-op Kanagawa, practiced measuring CC and MAC 10 times on a single subject to master the method of measurement. Then, the examiners measured CC and MAC twice on 5 subjects on different days, and each subject was measured by more than 2 examiners. From the data, intraclass correlation coefficients (ICC) for each examiner and the averages of the CC and MAC obtained from each subject were calculated. The examiners repeated the measurements if one of the following conditions were met: the ICC was <0.9; the value of the measurement was more than 1 cm above or below the average. The ICC of each examiner ranged from 0.90 to 1.00 for both the CC and MAC measurements. The averages of ICC were 0.97 ± 0.04 for CC and 0.96 ± 0.05 for MAC.

Memory impairment screening (MIS) [17] was used to confirm the credibility of the assessment with questionnaire. This test required subjects to recall 4 words and the score ranged from 0 to 8, with lower scores indicating poorer memory.

The 5 item geriatric depression scale (GDS5) was used to assess depressive symptoms [18]. The original version was translated into Japanese and established the reliability and validity [19]. It is scored from 0 to 5, with higher scores indicating more severe depression.

Functional limitation was assessed using the performance measure for activities of daily living 8 (PMADL-8) [20]. The PMADL-8 is composed of a list of 8 performance items potentially requiring daily physical activity in chronic heart failure patients and determines the extent to which patients currently experience difficulties in performing daily physical activity as evaluated by a four-category response scale. It is scored from 8 to 32, with higher scores indicating more severe functional limitations.

The participation scale was used to assess participation restriction [21]. The participation scale is composed of a list of 5 activity items, such as going out for a hobby or conversing/exchanging e-mails with family/friends. Response options for each question ranged from 1 to 4, and responses were determined by the frequency of the activities. It is scored from 5 to 20, with lower scores indicating more severe participation restriction.

We also assessed subjects' social support, which is received by interacting with others. Zimet et al. designed the original social support scale [22], and Iwasa et al. modified it to create a Japanese version, which consisted of 7 items [23]. We preliminarily surveyed healthy, community-dwelling elderly using the Japanese version of the questionnaire, and pared the original list of 7 items down to the following 3 items. (1) There is a special person who is around when I am in need. (2) My family really tries to help me. (3) I have friends with whom I can share my joys and sorrows. These three items were chosen because of the strong correlations between these 3 items and the other 4 items. We also confirmed that there is a strong correlation of the total score of these 3 items with the total of the 7 items score (unpublished data). The response options for each question ranged from 1 to 7, and responses were determined by the quality of the support. It is scored from 3 to 21, with higher scores indicating better support.

The mininutritional assessment (MNA) was used to assess nutritional status [24, 25]. MNA is composed of eighteen items, such as content of meals, BMI, internal use and midarm circumference (MAC). It is scored from 0 to 30, with lower scores indicating poorer nutritional condition.

### 2.3. Statistical Analysis

The data obtained from the subjects in care level 2 were compared with those in support levels and care level 1 using chi-square analyses and independent group *t* tests. Correlations between variables were assessed using Spearman's Rank correlation. Risk factors were defined as follows: (1) BMI <18.5 kg/m^2^, (2) grip strength ≤27 kg in males and ≤16 kg in females aged 65–74 and ≤20 kg in males and ≤ 12 kg in females aged 75 or older [5], (3) CC <31 cm [9], (4) GDS5 score ≥2 [18], and (5) MNA score ≤16 [24, 25].

For analysis of questionnaires, we excluded subjects with MIS scores ≤4, which indicated mild cognitive impairment level.

All data were analyzed using SPSS for Windows (version 16.0; SPSS, Tokyo, Japan). A *P* value <  0.05 was regarded as statistically significant.

## 3. Results

### 3.1. Study Population

A total of 417 subjects were surveyed (109 males and 308 females). Subject characteristics are presented in Table 1. We had planned to recruit the same numbers of subjects from care level 1 or lower and care level 2. However, that was difficult because the number of those who were difficult to communicate with or refused to cooperate with the survey was more than expected in care level 2. As a result, the number of subjects in care level 1 or lower doubled that in care level 2. The average age was 83 years old in each group. There were 83 males (29.9%) in care level 1 or lower and 26 males (18.7%) in care level 2. More than half of our subjects lived alone, and the ratio was higher in care level 1 or lower. One hundred and nine subjects (26.1% of the total) had MIS scores ≤4 and were excluded from the questionnaire analysis.

### 3.2. Prevalence of Risk Factors

The comparison of the prevalence of risk factors between care level 2 and care level 1 or lower were shown in Table 2. Care level 2 had higher prevalence of low grip strength and depression (GDS5 score ≥2) than care level 1 or lower. In contrast, there were no differences in the prevalence of CC <31 cm, BMI <18.5 kg/m^2^, and MNA ≤16 between the two groups. 

### 3.3. Correlations between Factors

The correlations between factors are shown in Table 3. Grip strength, CC, GDS5, and social support were correlated moderately or weakly with PMADL-8 and the participation scale, respectively. There were moderate correlations between MNA and BMI and between MNA and CC. Social support was negatively correlated with GDS5.

## 4. Discussion

The present study is the first study to compare the prevalence of possible objective risk parameters as well as questionnaires in those who were certified care level 2 or lower in Japanese LTC insurance system. The results of this study support our hypothesis that subjects in care level 2 had poorer grip strength, CC, and GDS5 values than those in care level 1 or lower.

Among our subjects, the prevalence of CC <31 cm, the cut-off value for predicting disability in female, was as high as 35%. Furthermore, grip strength was similar to those who were certified LTC eligibility level in previous studies [26]. Therefore, from the view point of muscle strength or mass, our subjects were frail and might be a representative sample of those who were certified same eligibility levels in Japanese LTC insurance system. 

The prevalence of malnutrition, indicated by MNA scores ≤16, was approximately 10%. This prevalence was 10 times as high as that in nondisabled elderly [27]. Similarly, 22.5% of the subjects in our study were underweight, with BMI values <18.5 kg/m^2^. This prevalence was two times greater than that in the independent community-dwelling elderly [28]. Low MNA score alone was reported as a risk factor for disability or death [12, 29, 30]. In addition, malnutrition and underweight may lead to sarcopenia, which is one of the primary causes of the onset of disability among the frail elderly population [31–33]. Thus, we confirmed that our subjects were at high risk for disability. However, there were no differences in the prevalence of MNA ≤16 and BMI <18.5 kg/m^2^ between care level 2 and care level 1 or lower. This was because nutritional status was not considered for certifying eligibility level in Japanese LTC insurance system [34]. Thus, malnutrition should be given more attention to prevent increasing eligibility level because the prevalence of malnutrition was high even in low eligibility levels. In addition to MNA and BMI, progressive weight loss should be taken into account because unintentional weight loss (5% in a half year or more) is one of the initial signs used as a screening for sarcopenia [31]. The future results of this study will reveal the relationship between malnutrition, being underweight or weight loss, and increasing eligibility level.

Depressive symptoms were related to eligibility level, activity limitations, and participation restrictions in the present study. In previous studies, depressive symptoms predicted disability, hospitalization, and death [35–37]. In contrast, social support were negatively correlated to depressive symptom, therefore, social support may be a countermeasure against the adverse impact of depressive symptoms on increasing eligibility level. This is because social support was shown to buffer the effects of negative life events or disease on mental health [38]. 

The prevalence of memory impairment was as high as 26%. This result highlighted the difficulty of using questionnaires to assess those who were certified eligibility level in LTC insurance system. The questionnaires for assessing depressive symptoms, nutritional status, and activity levels are useful tools to screen the risk for disability among community-dwelling elderly. However, memory impairment limits the credibility of the results obtained from these questionnaires. This result highlights the importance of objective parameters, such as grip strength, CC, or BMI, as the screening measure for increasing eligibility level in those who were certified LTC eligibility level.

We must describe potential limitations of the findings of the present study. Since this report was a cross-sectional study, it could not examine cause-effect relationships. Our ongoing prospective study will reveal risk factors for increasing eligibility level among Japanese home help service users. Another limitation was small sample of male subjects. Because of the gender differences in muscle strength or body composition, the analysis might be better to be carried out on the gender basis. Further study will need to examine the gender differences in the possible risk factors.

## 5. Conclusions

The findings of this study suggest that muscle strength, muscle mass, functional limitation, and depressive symptoms could be applied to stratify the high-risk subjects for increasing eligibility level. Our ongoing prospective cohort study will reveal the objective and subjective risk factors as well as their cut-off values for increasing eligibility level.

## Figures and Tables

**Table 1:** Comparison of characteristics between subjects in care level 1 or lower and those in care level 2.

	Care level 1 to less (*N* = 278, *N = 210)	Care level 2 (*N* = 139, *N = 98)	Statistics	*P* value
Age [yo]	82.94 ± 5.88	82.97 ± 7.26	*t* = −0.041	0.97
Female	195 (70.1%)	113 (81.3%)	*χ* ^2^ = 5.968	0.02
Living alone	177 (63.7%)	73 (52.5%)	*χ* ^2^ = 4.799	0.03
Service use				
Home bath service	0 (0%)	0 (0%)		
Visiting nurse	17 (6.1%)	28 (20.1%)	*χ* ^2^ = 18.944	0.00
Home rehabilitation	14 (5.0%)	12 (8.6%)	*χ* ^2^ = 2.051	0.20
Nursing home daycare	15 (5.4%)	23 (16.5%)	*χ* ^2^ = 13.913	<0.01
Health daycare	99 (35.6%)	64 (46.0%)	*χ* ^2^ = 4.235	0.04
Respite stay in a nursing home	3 (1.1%)	11 (7.9%)	*χ* ^2^ = 13.341	<0.01
Medical history				
Cancer	57 (20.5%)	27 (19.4%)	*χ* ^2^ = 0.067	0.90
Stroke	27 (9.7%)	32 (23.0%)	*χ* ^2^ = 11.366	<0.01
Heart disease	76 (27.3%)	37 (26.6%)	*χ* ^2^ = 0.024	0.91
Fracture of lower leg	52 (18.7%)	31 (22.3%)	*χ* ^2^ = 0.752	0.44
Compression fracture of the spine	33 (11.9%)	21 (15.1%)	*χ* ^2^ = 0.862	0.36
Rheumatoid arthritis	12 (4.3%)	7 (5.0%)	*χ* ^2^ = 0.11	0.81
BMI [kg/m^2^]	22.3 ± 4.2	21.3 ± 4.1	*t* = −1.19	0.24
Grip strength [kg]	19.1 ± 6.9	15.1 ± 6.7	*t* = 5.528	<0.01
CC [cm]	32.5 ± 3.4	31 ± 39	*t* = 3.795	<0.01
Memory impairment (MIS score ≦ 4)	68 (24.5%)	41 (29.5%)	*χ* ^2^ = 1.217	0.29
PMADL-8*	24.28 ± 4.877	27.8 ± 3.675	*t* = −6.986	<0.01
Participation scale*	13.93 ± 3.715	16.62 ± 2.646	*t* = −7.261	<0.01
Social support scale*	15.78 ± 4.09	15.51 ± 4.593	*t* = 0.503	0.62

BMI: body mass index; CC: calf circumference; MIS: memory impairment screen; PMADL-8: performance measure for activities of daily living 8. Values are mean ± SD or *n* (%).

*The number of subjects for questionnaires including PMADL-8, Participation scale, and Social support scale.

**Table 2:** Comparison of the number of those at risk for disability between care level 1 or lower and care level 2.

	Care level 1 to less (*N* = 278, *N = 210)	Care level 2 (*N* = 139, *N = 98)	*χ* ^2^	*P* value
BMI < 18.5 kg/m^2^	65 (23.4%)	29 (20.9%)	0.34	0.56
Low grip strength	56 (20.1%)	59 (42.4%)	23.1	<0.01
CC < 31 cm	92 (33.1%)	62 (44.6%)	0.02	0.29
GDS5* ≥ 2	87 (42.2%)	63 (64.3%)	12.9	<0.01
MNA* ≤ 16	21 (10.0%)	11 (11.2%)	0.11	0.84

Low grip strength was defined as ≤27 kg in males and 16 kg in females aged 65–74 and ≤20 kg in males and 12 kg in females aged 75 or older; BMI: body mass index; CC: calf circumference; GDS5: 5 item geriatric depression scale; MNA: mininutritional assessment. Values are *n* (%).

*The number of subjects for questionnaires including PMADL-8, Participation scale, and Social support scale.

**Table 3:** Spearman's Rank correlation.

	Age	BMI	Grip strength	CC	PMADL-8	Participation	Social support	MNA	GDS5
BMI	0.006								
Grip strength	−0.092	−0.017							
CC	−0.111*	0.028	0.354^†^						
PMADL-8	0.089	−0.021	−0.412^†^	−0.156^†^					
Participation	−0.136*	−0.009	0.273^†^	0.194^†^	−0.373^†^				
Social support	−0.035	−0.019	−0.046	0.044	−0.129*	0.214^†^			
MNA	−0.014	0.580^†^	0.048	0.130*	−0.013	0.037	0.060		
GDS5	0.087	−0.020	−0.160^†^	−0.168^†^	0.326^†^	−0.400^†^	−0.274^†^	−0.033	
MIS	−0.283^†^	−0.040	0.100*	0.117*	0.012	−0.069	0.053	−0.034	−0.028

*P < 0.05, ^†^ *P* < 0.01; BMI: body mass index; CC: calf circumference; MNA: mininutritional assessment; GDS5: 5 item geriatric depression scale; MIS: memory impairment screen.
